# Skin pH varies among bat species and seasons and between wild and captive bats

**DOI:** 10.1093/conphys/coab088

**Published:** 2021-12-06

**Authors:** Karen J Vanderwolf, Christopher J Kyle, Paul A Faure, Donald F McAlpine, Christina M Davy

**Affiliations:** Environmental and Life Sciences Program, Trent University, 1600 West Bank Dr., Peterborough, K9L 0G2, Ontario, Canada; Department of Natural History, New Brunswick Museum, 277 Douglas Ave, Saint John, E2K 1E5, New Brunswick, Canada; Environmental and Life Sciences Program, Trent University, 1600 West Bank Dr., Peterborough, K9L 0G2, Ontario, Canada; Forensic Science Department, Trent University, 1600 West Bank Dr, Peterborough, K9L 0G2, Ontario, Canada; Natural Resources DNA Profiling and Forensics Center, Trent University, 1600 West Bank Dr, Peterborough, K9L 0G2, Ontario, Canada; Department of Psychology, Neuroscience & Behaviour, McMaster University, 1280 Main Street West, Hamilton, L8S 4K1, Ontario, Canada; Department of Natural History, New Brunswick Museum, 277 Douglas Ave, Saint John, E2K 1E5, New Brunswick, Canada; Environmental and Life Sciences Program, Trent University, 1600 West Bank Dr., Peterborough, K9L 0G2, Ontario, Canada; Wildlife Research and Monitoring Section, Ontario Ministry of Northern Development, Mines, Natural Resources and Forestry, 1600 West Bank Dr, Peterborough, K9L 0G2, Ontario, Canada; Current affiliation: Department of Biology, Carleton University, 1125 Colonel By Drive, Ottawa, K1S 5B6, Ontario, Canada

**Keywords:** wildlife skin pH, white-nose syndrome, disease susceptibility, Cutaneous disease

## Abstract

Skin is a key aspect of the immune system in the defence against pathogens. Skin pH regulates the activity of enzymes produced both by hosts and by microbes on host skin, thus implicating pH in disease susceptibility. Skin pH varies inter- and intra-specifically and is influenced by a variety of intrinsic and extrinsic variables. Increased skin alkalinity is associated with a predisposition to cutaneous infections in humans and dogs, and inter-specific and inter-individual variation in skin pH is implicated in differential susceptibility to some skin diseases. The cutaneous pH of bats has not been characterized but is postulated to play a role in susceptibility to white-nose syndrome (WNS), a fungal infection that has decimated several Nearctic bat species. We used non-invasive probes to measure the pH of bat flight membranes in five species with differing susceptibility to WNS. Skin pH ranged from 4.67 to 8.59 and varied among bat species, geographic locations, body parts, age classes, sexes and seasons. Wild *Eptesicus fuscus* were consistently more acidic than wild *Myotis lucifugus, Myotis leibii* and *Perimyotis subflavus*. Juvenile bats had more acidic skin than adults during maternity season but did not differ during swarming. Male *M. lucifugus* were more acidic than females during maternity season, yet this trend reversed during swarming. Bat skin was more acidic in summer compared to winter, a pattern also reported in humans. Skin pH was more acidic in captive than wild *E. fuscus*, suggesting environmental impacts on skin pH. The pH of roosting substrates affects skin pH in captive bats and may partially explain seasonal patterns in wild bats that use different roost types across seasons. Future research on the influence of pH on microbial pathogenic factors and skin barrier function may provide valuable insights on new therapeutic targets for treating bat skin conditions.

## Introduction

Skin is a complex physical barrier and chemical landscape representing one of the first lines of defence that hosts have against pathogens (Elias, 2005; Byrd *et al.*, 2018). Despite direct environmental exposure to microbiota, skin is largely unsuitable for microbial colonization, unlike mucosal surfaces (Chen *et al.* 2018). Physiological properties of the skin can affect innate immune function in addition to influencing the growth of microbes (Diamond *et al.*, 2009; Jantsch *et al.*, 2015). Skin surface defences against microbial invasion include the combined effects of desiccation, epidermal desquamation, acidic pH, nutrient limitations, commensal microbes, antimicrobial lipids (sebum), and antimicrobial peptides (Harder *et al.* 2013; Naik *et al.* 2012). Disruption of these defences can affect susceptibility to cutaneous diseases (Harder *et al.* 2013; Naik *et al*. 2012).

Cutaneous pH may alter pathogen virulence or host susceptibility because pH affects enzyme production, activation and efficiency in hosts as well as their commensal microbes and pathogens (Elias, 2005). The pH of skin influences at least four key epidermal functions: permeability barrier homeostasis, integrity/cohesion (desquamation), initiation of inflammation, and antimicrobial defence (Hachem *et al.*, 2003; Elias, 2005). Recovery of human and laboratory mice skin barrier function after injury proceeds normally at an acidic pH (<6 pH), but is delayed at a neutral pH (i.e. 7–7.4 pH) as a result of impaired post-secretory processing of extracellular lipids in the lower stratum corneum by pH-dependent enzymes (Behne *et al.*, 2002; Proksch and Neumann, 2019). Alkaline (basic) skin pH can increase virulence of several fungal pathogens by facilitating penetration into host surfaces and evasion of immune responses (Vylkova, 2017). Attempts to induce *Candida albicans* (pathogenic yeast) lesions were more successful on human skin alkalized to 6.0 pH with topical products compared to unaltered skin at 4.5 pH (Runeman *et al.*, 2000). This pattern was not caused by inhibited growth of *C. albicans*, but instead was thought to be due to pH dependence of either the yeast’s virulence capacity or modulations of the host’s defences (Runeman *et al.*, 2000). Increased skin alkalinity in humans, laboratory mice, and dogs is associated with a predisposition to cutaneous infections such as bacterial pyoderma, multiple types of dermatitis, acne, eczema, candidiasis, tinea, and diaper rash (Chikakane and Takahashi, 1995; Matousek and Campbell, 2002; Matousek *et al.*, 2003; Hatano *et al.*, 2009). These findings suggest that skin pH may also be important in cutaneous wildlife diseases such as amphibian chytridiomycosis and bat white-nose syndrome (WNS), both of which have devastated some species but not others (Fisher *et al.*, 2016). Indeed, *Batrachochytrium dendrobatidis* infection load, the cause of chytridiomycosis, was positively correlated with pH on the ventral, but not the dorsal, skin of frogs, which may be a cause or a consequence of infection (Woodhams *et al.*, 2012).

Skin pH is genetically determined to a degree, but is also affected by behaviour and environment (Sakuma and Maibach, 2012). Factors influencing skin pH include the following: (i) endogenous factors such as age, anatomical location, genetic predisposition, amount of melanin in skin, glandular secretions (sebaceous, apocrine, eccrine) and moisture; and (2) exogenous factors such as topical products, occlusive dressings and skin irritants (e.g. various chemicals; Matousek and Campbell, 2002; Schmid-Wendtner and Korting, 2006). Mouse skin is largely acidified by endogenous agents, such as the sodium-proton antiporter (NHE1) and secretory phospholipase A_2_ (sPLA_2_)-mediated extracellular generation of free fatty acids from phospholipids (Behne *et al.*, 2002; Fluhr *et al.*, 2004a). Research on humans, laboratory and domestic mammals show that skin pH varies with season (human skin most acidic in July), body part, sex, age, species and breed (Byrd *et al.*, 2018; Chikakane and Takahashi, 1995; Matousek and Campbell, 2002; Meyer and Neurand, 1991). Skin pH of wildlife has rarely been studied (Supplementary Table S1), but does include data for various bird species and naked mole rats (*Heterocephalus glaber*) in zoos (Bartels *et al.*, 1991; Menon *et al.*, 2019) and amphibians and fish in laboratories (Tsui *et al.*, 2002; Litwiller *et al.*, 2006; Woodhams *et al.*, 2012; Barnhart *et al.*, 2020). Skin pH has not yet been quantified in free-ranging populations but may be an important component in assessing both inter- and intra-specific responses to infectious pathogens.

Skin diseases of wildlife have received increasing attention over the past few decades (Fisher *et al.*, 2016). The best-known skin disease of bats is WNS, a cutaneous infection caused by the fungal pathogen *Pseudogymnoascus destructans* that damages flight membranes during hibernation and can lead to starvation, dehydration, and death (Lorch *et al.*, 2011; Cryan *et al.*, 2013). The disease varies seasonally and variation in host susceptibility has been documented both among and within species (Frank *et al.*, 2014; Langwig *et al*. 2012; Turner *et al.*, 2011). Some Nearctic bat species have experienced catastrophic population declines due to WNS and are now listed as endangered (Solari, 2018). Previous research on the ability of *P. destructans* to use various nutrient sources, secrete enzymes, and interact with other microbes conducted experiments at various pH levels, without knowing the pH of bat skin (Beekman *et al.*, 2018; Donaldson *et al.*, 2018; Gabriel *et al.*, 2019; Vanderwolf *et al.*, 2021). Cultures of *P. destructans* grow *in vitro* from 4 to 11 pH (Raudabaugh and Miller, 2013; Vanderwolf *et al.*, 2021), although a carboxypeptidase enzyme produced by the fungus *in vitro* was most active at 3–5 pH compared to 6.5–8.5 pH (Beekman *et al.*, 2018). Cultures of *P. destructans* alkalinize some growth media *in vitro* (e.g. 5.6–7.9 pH) (Veselská *et al.*, 2020), but it is unknown if the fungus alkalinizes bat skin. Prior to WNS, skin diseases were not commonly reported in bats, although dermatophytes are known to grow on bat skin (Simpson *et al.*, 2013; Lorch *et al.*, 2015; McAlpine *et al.*, 2016) and dermatitis has been documented (Goodnight, 2015; Fountain *et al.*, 2017, 2019). A global survey of captive bats found that some species are more frequently reported with skin diseases compared to others, and some skin lesions show seasonal patterns with increased frequency in the winter for bats exposed to outdoor temperatures (Fountain *et al.*, 2017).

Given the strong link between skin chemistry and susceptibility to cutaneous diseases demonstrated in humans and domestic mammals, variation in skin chemistry may partly explain why bats vary in susceptibility to skin diseases such as WNS. Inter- and intra-specific or seasonal variation in bat skin pH may partially explain corresponding variation in cutaneous microbiomes and responses to pathogens. We measured the skin pH of 5 bat species at 32 locations across eastern Canada over 1 year to determine how flight membrane pH differs with species, season, body part, sex, age-class, geographic location, and pH of roosting substrates. Skin pH of humans varies among body parts and seasons (Abe *et al.*, 1980; Schmid-Wendtner and Korting, 2006; Wan *et al.*, 2014), and we hypothesized that similar mechanisms apply to bats, predicting that we would observe the most acidic skin pH in summer months. Previous research on humans and domestic animals found sex-based variation in skin pH and therefore we predicted there would be variation in skin pH between sexes in bats. However, the direction of the sex effect varied among species and studies (Jenkinson and Mabon, 1973; Ruedisueli *et al.*, 1998; Giacomoni *et al.*, 2009; Szczepanik *et al.*, 2011), so we could not predict the direction of the effect in bats. Finally, while we could not make directional predictions about site-specific variation in bat skin pH, we expected that roost site characteristics might affect bat skin pH, predicting that skin pH might vary among capture locations. Data on the skin pH of bats will inform future research into the functionality of enzymes on the skin surface. Our interest in this topic stems from the potential link between skin chemistry and disease susceptibility. The bats we measured in this study have all survived multiple years with WNS, meaning that our study populations of ‘susceptible’ *M. lucifugus* have already undergone selection for tolerance or resistance to WNS (Donaldson *et al.*, 2017; Cheng *et al.*, 2019; Auteri and Knowles, 2020). Therefore, we did not attempt to correlate skin pH directly with species’ susceptibility to WNS. Instead, our study provides a baseline for further work on disease susceptibility and potential treatments for skin diseases of bats.

## Methods

**Figure 1:**
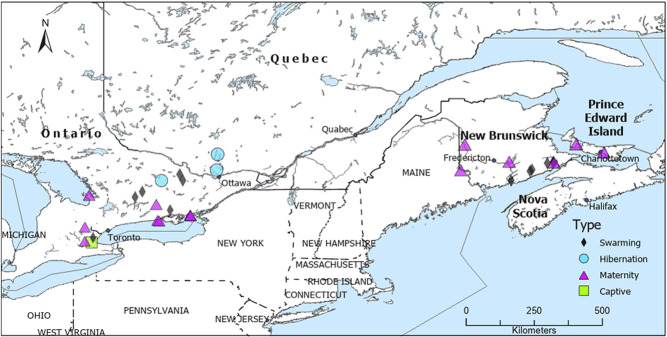
Sites where we measured the skin pH of bats at maternity (*n* = 15 sites; *n* = 270 individual wild bats), swarming (*n* = 13; *n* = 389) and hibernation sites (*n* = 3; *n* = 51) in 2019–2020.

We caught wild bats in eastern Canada at (i) maternity colonies, where bats give birth and raise pups (May–mid-July 2019); (ii) swarming sites, where bats congregate and mate at potential hibernacula such as caves and mines (mid-July–October 2019); and (iii) hibernation sites, where bats overwinter in underground structures (February 2020) (Fig. 1). Bats at maternity and swarming sites were caught using mist nets and harp traps, while bats at hibernation sites (caves and mines) were caught by hand from the walls and ceilings. Maternity colonies were in bat boxes, attics, and the siding of buildings. Bats were caught outside caves and mines during swarming season. There was temporal overlap of measurements taken of wild bats in Atlantic Canada and Ontario during maternity and swarming seasons. Wild bats were only measured in February during the hibernation period to minimize disturbance, and gloves were changed between processing each bat to minimize microbial transfer. We recorded the species, sex, weight, and age (juvenile or adult) of each bat (OMNRF WACC authorization #19–394; Trent University animal care authorization 26 117, New Brunswick Species at Risk permit #SAR19-014). Species included *Eptesicus fuscus*, *Myotis lucifugus*, *Myotis leibii, Myotis septentrionalis*, and *Perimyotis subflavus.* We distinguished young-of-the-year from adults by examining the degree of fusion of the epiphyseal growth plates of the phalanges in July and August (Kunz and Anthony, 1982); however, some young-of-the-year were likely classified as adults during swarming season. We were unable to differentiate age classes further in our study, but follow-up work could also record whether testes were descended, to further separate young-of-the-year from adults. Bats were released on site after we completed measurements. Field work was only conducted on nights with no rain in the interests of bat welfare.

**Figure 2:**
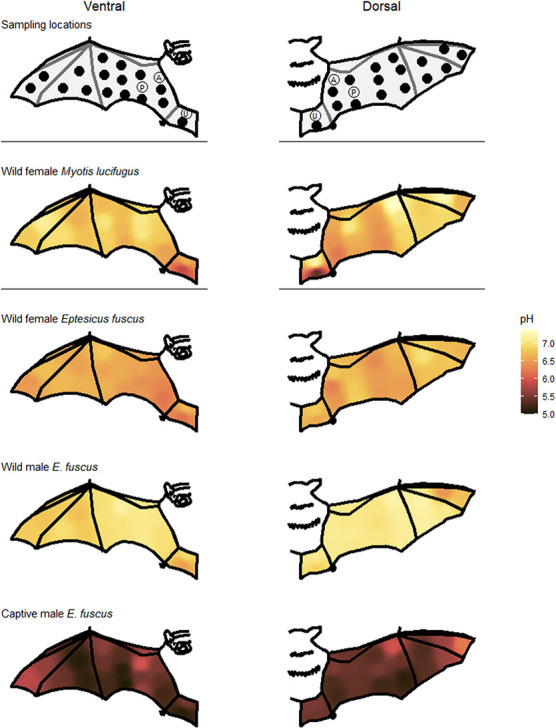
Schematic views of the right wing and tail membrane indicating where we measured skin pH. All 38 measurements were taken from four individual bats while ‘P’ (ventral and dorsal plagiopatagium), ‘A’ (ventral and dorsal arm) and ‘U’ (ventral and dorsal uropatagium) were taken from all bats. Heat maps illustrate skin pH measurements taken from the ventral (left; 19 skin sites per bat) and dorsal (right; 19 skin sites per bat) flight membranes of bats caught in Ontario 2019. *Myotis lucifugus* and captive *E. fuscus* were measured in June and the two wild *E. fuscus* were measured in May.

We measured skin pH using a pH meter (PH905; Courage and Khazaka Electronic GmbH, Mathias-Brüggen-Str. 91 50829 Köln, Germany) that attaches to a multiprobe adapter system (MPA2; Courage and Khazaka Electronic). The probe measures surface pH and does not penetrate the skin. The diameter of the sensor was 1 cm. We took three consecutive measurements per skin site with the pH meter and used the mean as the final value. We repeated measurements if the three measurements from one skin site differed by more than 0.2 pH, as large variation indicates the probe was held incorrectly. Since skin pH varies among body parts in humans (Schmid-Wendtner and Korting, 2006), we quantified fine-scale variation in skin pH across the flight membrane by taking measurements of 38 sections (in a grid pattern) on the right wing and tail membrane from a subset of bats (Fig. 2; 4 individuals). Based on these initial results (Fig. 2), and to standardize measurements among individual bats and investigate variation in pH among body parts, we subsequently took three measurements (‘arm’, ‘plagiopatagium’ and ‘uropatagium’; Fig. 2) on the dorsal side and three measurements on the ventral side of the right wing and tail membrane. We stored the end of the pH probe in KOH and washed it in distilled water between each set of measurements, as recommended by the manufacturer. We calibrated the pH probe every day for the first month and thereafter once a week with 4 and 7 pH buffers, exceeding the manufacturer recommendation of calibration every 3 weeks.

Initially, we also attempted to measure the amount of sebum on the surface of bat flight membranes with a sebumeter (SM815; Courage and Khazaka Electronic GmbH, Mathias-Brüggen-Str. 9 150 829 Köln, Germany). However, this probe was designed for use on humans and, from our initial observations, was not sensitive enough to detect small amounts of sebum on bat flight membranes. Many of our measurements of bat skin using the sebumeter were zero. Non-zero values were not reproducible and appeared to be affected by the presence of urine on the skin.

To investigate temporal variation in skin pH, we measured an *E. fuscus* captive research colony at McMaster University (Hamilton, Ontario, Canada) monthly from April 2019 to March 2020. The colony has two living areas: an ‘Established’ side and a ‘Quarantine’ side. The Established side houses bats that have passed quarantine, whereas the Quarantine side houses recently captured wild bats that stay in quarantine for a minimum of 3 months after arriving, as well as bats that have been in the colony for months or years but are being used in current experiments. Bats in the Established colony have year-round access to an outdoor flying area (Skrinyer *et al.*, 2017). Bats on both sides of the colony have constant access to water, meal worms (*Tenebrio molitor*, Reptile Feeders, Norwood, Ontario, Canada) and space allowing them to fly. The colony temperature and lighting vary with ambient conditions; however, both indoor living areas are buffered from ambient temperatures, particularly in the winter (Supplementary Fig. S1). Daily maximum and minimum temperature and humidity in the captive colony was measured with an Acurite indoor/outdoor digital thermometer and hygrometer (model # 00219CA). The captive bats typically roost in between and behind layers of cotton bath towels folded in half and hung on the colony walls, and some bats roosted inside two wooden structures. To investigate possible influences of roost pH on skin pH we also measured the pH of each layer of the four towels (1 outside layer, 3 inner layers of each towel; 3 measurements for each layer) monthly from December–March 2019. We measured the inside surface pH of the wooden roosts once in February 2019.

We conducted a literature review on the skin pH of animals to put our data in the context of previous studies. We located papers using the internet search engines Thomson Reuters’ ISI Web of Science and Google Scholar, as well as by scanning bibliographies of relevant papers, on 3 May 2021 using the keywords ‘wildlife “skin pH”’ and ‘animal “skin pH”’. Conference abstracts and posters were excluded and only studies on intact animals were included (i.e. *in vitro* studies of tissue samples were excluded).

### Data analysis

Unless stated otherwise, data are presented as the mean ± standard deviation (SD). We performed all statistics in R (R Core Team, 2020). We constructed all graphs using ggplot2 (Wickham, 2016). Data used to construct Fig. 2 were interpolated using the function ‘idw’ in the gstat package (Graler *et al.*, 2016) in addition to using ggplot2, raster, scico and sf packages (Wickham, 2016; Pebesma, 2018; Pedersen and Crameri, 2020; Hijmans, 2021). We used linear mixed effects models (package ‘lme4’; Bates *et al.*, 2015) to determine which variables affected bat skin pH in three separate models for the capture seasons: maternity (May–July), swarming (August–October) and hibernation (November–April). We set the individual bat as a random effect in each model (six measurements taken per bat) to control for inter-individual variation. Fixed effects potentially affecting skin pH included intrinsic (sex, species, age, body part, flight membrane surface) and extrinsic factors (day of year and site). ‘Membrane surface’ refers to the dorsal and ventral sides of the flight membranes, and ‘body part’ refers to the three flight membranes that were measured: arm, plagiopatagium and uropatagium. We did not include age (juvenile, adult) in the hibernation model because young-of-the-year cannot be differentiated from adults during winter. We also did not include day-of-year in the hibernation model because the skin pH of wild bats during the hibernation period were measured over a 10-day period in February. Additionally, we used generalized additive mixed models with individual bat as a random effect using the packages ‘mgcv’ and ‘MuMIn’ (Wood, 2017; Barton, 2019) to determine the impact of the fixed effects previously listed on skin pH for three species (*E. fuscus*, both captive and wild caught; *M. lucifugus*; and *M. leibii*). We applied a smoothing factor to day-of-year for each bat species. We added maximum and minimum temperature and relative humidity (on the measurement day) as fixed effects to the model for captive *E. fuscus*. We excluded *M. septentrionalis* from statistical analyses given low sample size (4 individuals). We used a linear mixed effect model for *P. subflavus* because this species was sampled over a limited time span. We used the function AICtab (package bbmle) (Bolker and Team RDC, 2017) to compare Akaike information criteria (AIC) values to determine the best model. Including ‘region’ (Ontario, Quebec, region 1; Maritime provinces, region 2) as a fixed effect in the models for maternity season, swarming season, wild *E. fuscus*, and *M. lucifugus* did not improve the models. Models with ‘region’ in place of ‘site’ were inferior. Region was not included in models for hibernation, *P. subflavus*, captive *E. fuscus*, and *M. leibii* because measurements were obtained in only one region. We compared the skin pH of captive and wild *E. fuscus* with a generalized additive mixed model (smoothing factor applied to day-of-year), with captive status, day-of-year, sex, body part, and membrane surface as fixed effects and individual bat as a random effect. We tested for intra-individual associations of skin pH among the six body parts measured using the captive colony dataset with a repeated measures correlation in the package ‘rmcorr’ (Bakdash and Marusich, 2020). We tested whether the rank order of captive individual bats was consistent across 12 months of sampling by calculating the intraclass correlation coefficient using the package ‘irr’ (Gamer *et al.*, 2019) with a one-way model, inter-rater agreement and the mean skin pH of the six body parts for each individual in each month.

## Results

**Figure 3:**
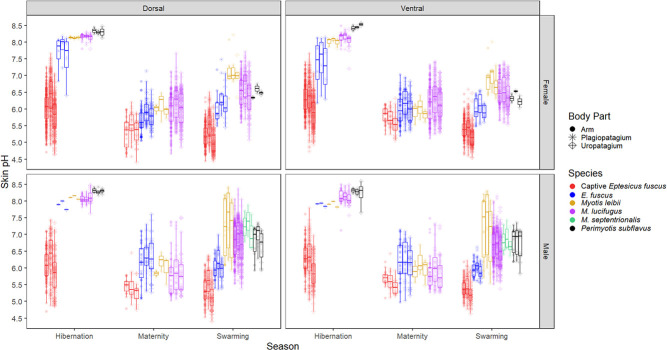
Box plots of flight membrane pH of captive *E. fuscus* and wild bats measured in Ontario, Quebec, New Brunswick and Prince Edward Island, Canada, across three activity seasons: maternity (May–July), swarming (August–October) and hibernation (November–April for captive bats, wild bats only measured in February).

We measured 710 wild bats comprising five species (Supplementary Table S2). The range in skin pH was 4.67–8.50 for *M. lucifugus* (*n* = 528 individual bats), 5.48–8.42 for *M. leibii* (*n* = 28), 6.36–7.88 for *M. septentrionalis* (*n* = 4), 5.83–8.59 for *P. subflavus* (*n* = 19), 4.97–8.17 for wild *E. fuscus* (*n* = 131), and 4.40–7.80 for captive *E. fuscus* (*n* = 678 measuring sessions for 126 individual bats). Skin pH varied among species and on average *E. fuscus* was the most acidic across all three seasons (maternity, swarming, and hibernation), although there was no significant pH difference between wild *E. fuscus* and *M. leibii* during hibernation (Table 1; Fig. 3).

**Table 1:** Results of linear mixed effects models with variables explaining flight membrane pH of wild bats at maternity, swarming and hibernation sites

Variable	Maternity	Swarming	Hibernation	Captive *E. fuscus*	Wild *E. fuscus*	*M. lucifugus*	*M. leibii*	*P. subflavus*
R^2^	m = 0.66, c = 0.80	m = 0.67, c = 0.90	m = 0.44, c = 0.81	0.65	0.67	0.82	0.95	m = 0.95, c = 0.97
Site	F_14_ = 31.3, *P* < 2.2e-16	F_12_ = 17.4, *P* < 2.2e-16	F_2_ = 6.3, *P* = 0.004	NA	F_13_ = 6.9, *P* = 9.4e-13	F_25_ = 13.6, *P* < 2e-16	**F**_**6**_ **= 210.2, *P* < 2e-16**	**F**_**4**_ **= 180.0, *P* = 4.2e-13**
Species	F_2_ = 31.4, *P* = 7.2e-13	F_4_ = 18.8, *P* = 4.3e-14	**F**_**3**_ **= 11.6, *P* = 9.4e-6**	NA	NA	NA	NA	NA
Day of year	F_1_ = 48.5, *P* = 8.2e-12	F_1_ = 47.1, *P* = 2.8e-11	NA	**F**_**11.0**_ **= 651.3, *P* < 2e-16**	F_8.4_ = 9.1, *P* = 1.2e-12	F_8.4_ = 11.6, *P* = 2.7e-11	**F**_**1.0**_ **= 19.6, *P* = 1.8e-5**	D
Sex	F_1_ = 19.5, *P* = 1.4e-5	F_1_ = 4.1, *P* = 0.044	F_1_ = 0.1, *P* = 0.703	F_1_ = 7.2, *P* = 0.007	F_1_ = 14.7, *P* = 1.3e-4	F_1_ = 34.2, *P* = 5.6e-9	D	D
Membrane surface (dorsal/ventral)	**F**_**1**_ **= 48.9, *P* = 4.2e-12**	**F**_**1**_ **= 225.7, *P* < 2.2e-16**	F_1_ = 5.5, *P* = 0.019	F_1_ = 178.9, *P* < 2e-16	**F**_**1**_ **= 49.0, *P* = 5.6e-12**	F_1_ = 66.9, *P* = 4.2e-16	F_1_ = 2.4, *P* = 0.123	D
Body part	**F**_**2**_ **= 101.2, *P* < 2.2e-16**	**F**_**2**_ **= 466.4, *P* < 2.2e-16**	F_2_ = 5.3, *P* = 0.006	**F**_**2**_ **= 456.6, *P* < 2e-16**	F_2_ = 35.0, *P* = 3.0e-15	**F**_**2**_ **= 161.5, *P* < 2e-16**	F_2_ = 14.8, *P* = 1.4e-6	**F**_**2**_ **= 10.9, *P* = 5.4e-5**
Age (adult/juvenile)	F_1_ = 24.9, *P* = 1.1e-6	D	NA	NA	F_1_ = 5.0, *P* = 0.025	F_1_ = 7.1, *P* = 0.008	NA	NA
Body part*wing surface	F_2_ = 7.4, *P* = 6.2e-4	F_2_ = 36.2, *P* = 3.6e-16	F_2_ = 3.4, *P* = 0.036	F_2_ = 58.2, *P* < 2e-16	F_2_ = 6.3, *P* = 0.002	F_2_ = 18.5, *P* = 1.0e-8	F_2_ = 2.5, *P* = 0.09	D
Sex*day of year	F_1_ = 17.3, *P* = 4.0e-5	D	NA	NI	NI	NI	NI	D
Body part*day of year	D	D	NA	NI	NI	NI	NI	D
Wing surface*day of year	F_1_ = 38.2, *P* = 8.5e-10	D	NA	NI	NI	NI	NI	D
Sex*body part	D	D	D	D	D	F_2_ = 5.4, *P* = 0.005	D	D
Sex*wing surface	F_1_ = 35.3, *P* = 3.6e-9	F_1_ = 30.2, *P* = 4.4e-8	**F**_**1**_ **= 6.7, *P* = 0.010**	F_1_ = 11.7, *P* = 6.5e-4	**F**_**1**_ **= 37.4, *P* = 1.5e-9**	**F**_**1**_ **= 199.6, *P* < 2e-16**	D	D

We performed generalized additive mixed models for each bat species, except for *P. subflavus* where we used a linear mixed effect model. Maximum and minimum temperature and relative humidity were dropped from the best model for captive *E. fuscus* and are not shown. The marginal (m) and conditional (c) R^2^ are reported for linear mixed effect models, and the *F*-value with degrees of freedom and *P*-value are reported for each variable in each model. The two variables explaining the most variance in each model are in boldface type.

NA, not applicable; D, dropped from the best model; NI, not included in the model.

**Figure 4:**
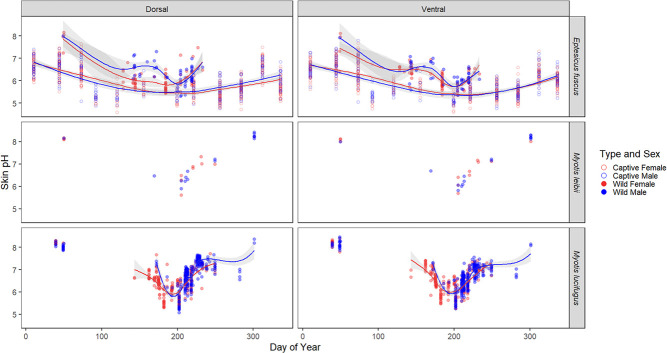
Plagiopatagium flight membrane pH of *E. fuscus* (captive and wild) and wild *M. leibii* and *M. lucifugus* over time (data from all provinces). Sample sizes are listed in Supplementary Table S2. Lines indicating the mean pH (95% confidence intervals in grey shading) were predicted using the loess method. Seasonal patterns in *M. septentrionalis* and *P. subflavus* could not be assessed due to low sample sizes.

Bats were most acidic in July in both the captive *E. fuscus* colony (5.1 ± 0.3 pH in July) and wild bats (6.0 ± 0.5 pH in July for all species at all sites), but note that wild bats were not measured from November–April, except for February (Fig. 4). Although wild bats were measured at multiple sites, the SD among wild bats was similar to that observed in the captive colony (0.5 vs. 0.3). This suggests that the time series of the captive bats’ skin pH provides a meaningful benchmark for temporal trends in skin pH of wild bats, despite colony-specific variation that may be associated with different roosting substrates or diet. The skin pH of wild bats had large seasonal variations, while seasonal patterns in the captive *E. fuscus* colony were more attenuated (Fig. 4). Skin pH significantly decreased over the maternity season and increased over the swarming season in both wild and captive bats (Table 1; Fig. 4). The skin pH of captive *E. fuscus* gradually increased from the beginning of hibernation season, peaked in February (6.4 ± 0.5 pH) and then gradually decreased towards spring (Fig. 4). Changes in skin pH over the hibernation season could not be assessed for hibernating wild bats since they were exclusively measured in February (8.1 ± 0.3 pH for four species of wild bats in February).

**Figure 5:**
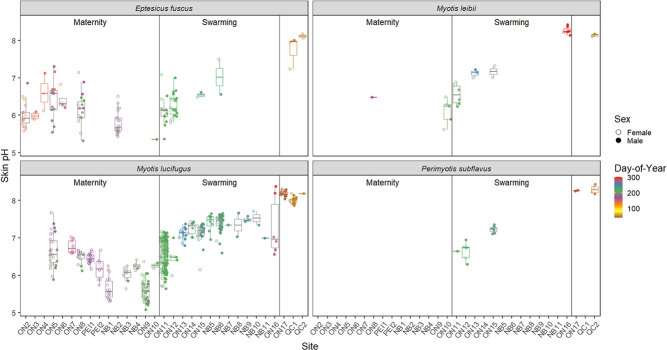
Dorsal plagiopatagium pH of wild bats at each geographical site. Sites are listed with their provincial abbreviation: ON, Ontario; NB, New Brunswick; PEI, Prince Edward Island; and QC, Quebec. Sites ON2–ON10, PEI1–PEI2 and NB1–NB4 were measured during the maternity season. Sites ON11–ON16 and NB5–NB11 were measured during swarming season. Sites ON17, QC1 and QC2 were measured during the hibernation season.

Geographic site also influenced skin pH, although its effect was dependent on the time of year each site was sampled (Table 1; Fig. 5). For example, we measured *M. lucifugus* at site ON16 early and late in the swarming season and skin pH increased over this period (Supplementary Fig. S2). Similarly, we measured both *E. fuscus* and *M. lucifugus* at sites ON5 and ON8 early and late in maternity season and skin pH decreased over this time (Supplementary Fig. S2).

**Figure 6:**
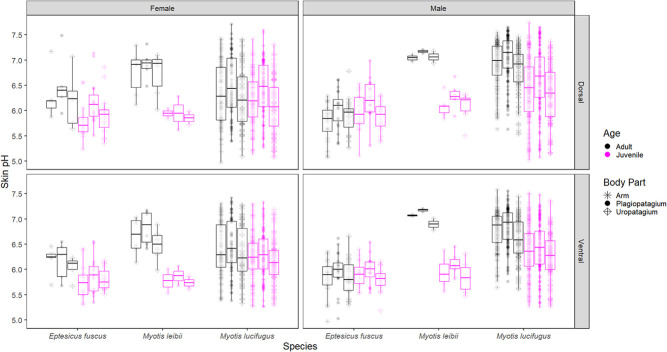
Wild volant juvenile and adult bats caught in Ontario and New Brunswick from day-of-year 186–250 (i.e. the first to last days that juveniles were caught). Note: we did not catch juvenile *P. subflavus* of either sex.

There was no significant difference in skin pH between wild juvenile and adult bats during swarming, but juveniles were more acidic than adults in the maternity season model and in the *E. fuscus* and *M. lucifugus* species models (Table 1; Fig. 6). Although all juveniles included in the statistical analysis were volant, two adult females were caught carrying non-volant pups during the maternity season in New Brunswick. A female *E. fuscus* caught 3 July 2019 carrying a furless pup had a mean of 5.6 pH (range: 5.45–5.64) for the six standard skin measurements, while the pup had a mean of 5.15 pH on its back. A female *M. lucifugus* caught 11 July 2019 was carrying a furred male pup, which measured 6.2 pH on the lower back, while the mother’s mean for the six standard skin measurements was 6.1 pH (range: 6.04–6.22).

Wing skin of male *M. lucifugus* and captive *E. fuscus* were more acidic than females during maternity season (and during late hibernation in captive bats), but this trend reversed during swarming (and early hibernation in captive bats) (Fig. 4). Wild *E. fuscus* males were more alkaline than females during maternity season. There were no sex differences in skin pH from wild bats during hibernation or in *M. leibii* and *P. subflavus* (Table 1; Fig. 3).

**Figure 7:**
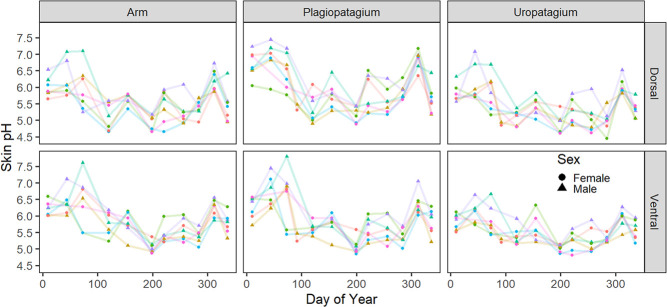
Skin pH from seven bats in the captive *E. fuscus* colony in Hamilton, Ontario, that we measured 10–11 times each in 2019–2020. Each colour indicates an individual bat. The availability of individuals in the colony varied over time; the individuals that were measured most frequently are depicted.

The pH of individual bats was not constant over time. The skin pH of five wild bats caught twice and two wild bats caught three times varied over time, with different patterns among individuals (Supplementary Fig. S3). The skin pH of individual captive *E. fuscus* also varied temporally, and there was agreement among months in the rank order of individual bats with respect to mean skin pH (F_1,22_ = 0.478, *P* = 0.497), implying the impact of external factors (Fig. 7).

In wild bats, mean pH range among body parts of an individual was 0.60 ± 0.28 (range: 0.11–1.78; Supplementary Fig. S4), while in captive bats it was 0.78 ± 0.27 (range: 0.22–1.84). Dorsal flight membranes were more acidic than their ventral surfaces during maternity season, particularly in females (Table 1; Fig. 3). This pattern reversed during swarming and hibernation seasons as the ventral surface was more acidic in wild bats, particularly for males during swarming. Wing sites closest to the body were more acidic than those further from the body, and the ventral uropatagium was particularly acidic (Fig. 2). The plagiopatagium was the most alkaline flight membrane and the uropatagium was the most acidic during swarming in all bat species except wild *E. fuscus*. The arm was most acidic in wild *E. fuscus* and during maternity season. Skin pH did not differ among body parts during hibernation in wild bats, although differences persisted in captive *E. fuscus* (Fig. 3). Skin pH for the six standard flight membrane locations were highly correlated with each other within an individual over time in captive bats (Table 2).

**Table 2:** Repeated measures correlations for the six flight membrane sites measured in captive *E. fuscus*

Variable	Dorsal plagiopatagium	Dorsal arm	Dorsal uropatagium	Ventral plagiopatagium	Ventral arm	Ventral uropatagium
Dorsal plagiopatagium	1					
Dorsal arm	**0.90**, 0.88:0.91, 1.07e-215	1				
Dorsal uropatagium	**0.84**, 0.82:0.87, 9.9e-162	**0.91**, 0.90:0.93, 5.9e-233	1			
Ventral plagiopatagium	**0.82**, 0.80:0.85, 4.4e-148	**0.85**, 0.83:0.87, 4.7e-167	**0.85**, 0.83:0.87, 5.3e-169	1		
Ventral arm	**0.81**, 0.78:0.84, 5.6e-141	**0.84**, 0.82:0.86, 1.4e-160	**0.84**, 0.81:0.86, 4.7e-158	**0.95**, 0.94:0.96, 1.7e-304	1	
Ventral uropatagium	**0.79**, 0.75:0.82, 4.3e-162	**0.86**, 0.84:0.88, 1.7e-178	**0.89**, 0.88:0.91, 4.7e-208	**0.86**, 0.84:0.88, 2.4e-173	**0.89**, 0.87:0.91, 5.1e-204	1

Mean correlations (R_rm_; in boldface), the 95% confidence interval and *P*-value are listed for each pairwise comparison. The degrees of freedom for each comparison was 591.

Captive *E. fuscus* (model estimate = 5.6 ± 0.02, *P* < 2e-16) had more acidic skin than wild *E. fuscus* (model estimate = 6.4 ± 0.04, *P* < 2e-16; Fig. 4). The maximum and minimum temperature and relative humidity were dropped as explanatory variables from the best model for the skin pH of captive *E. fuscus*, indicating they explained little to no variation in skin pH. The pH of the four towels measured over four months in the captive colony was 6.0 ± 0.6 for the outer layer and 5.6 ± 0.4 for the three inner layers (range: 4.5 pH for the inner layers to 6.89 pH for the outer layer). The inside lid and walls of the wooden roost structure in the established captive colony were 6.7 pH and 6.6 pH, respectively. In contrast, the inside lid and walls of a similar wooden roost structure in the relatively little-used quarantine side of the colony measured 7.3 pH and 8.0 pH, respectively. The roosting towels in the established side of the captive colony were replaced with clean towels twice over the study period: first, a month before measurements were recorded in June 2019 and again a week before the November 2019 measurements. These towel changes correlated with an increase in skin pH of individual captive bats (Fig. 7).

A summary of previous literature on the skin pH of non-human vertebrates is presented in Supplementary Table S1. Several methodological details were sometimes missing from papers, particularly the time of year measurements were taken. Previous studies exclusively measured captive or domestic animals, with dogs and laboratory mice the most commonly studied.

## Discussion

We investigated variation in the skin pH of bats to provide a baseline for future research on the association between skin chemistry and cutaneous infection in bats and other wildlife, exploring how skin pH varied among species, body part, season, sex, age class, and sampling location. Among the species we measured, *E. fuscus* had the most acidic skin and *M. septentrionalis* had the most alkaline skin (Fig. 3). While we could not explicitly test the link between skin pH and WNS susceptibility, it is intriguing that the five species’ skin pH fell along the same spectrum as their predicted susceptibility to WNS. Skin pH also varied between the sexes, by season (most acidic in July), and among body parts, which is consistent with previous studies on the skin pH of humans and domestic mammals (Byrd *et al.*, 2018; Chikakane and Takahashi, 1995; Matousek and Campbell, 2002; Meyer and Neurand, 1991). The direction of the sex effect changed between the maternity and swarming season for *M. lucifugus* and *E. fuscus*. The pH of bat flight membranes also varied by age class and sampling location (Table 1).

Our study is the first to measure skin pH in free-ranging vertebrates. Comparing our results with previous research on skin pH is difficult because seasonal variation has only been studied in humans, and most studies did not report which months the measurements were taken (Supplementary Table S1). Additionally, some studies measured the fur/hair of animals instead of directly measuring the skin, although the fur/hair was shaved or clipped prior to measurement in some investigations. Nevertheless, our measurements of bat skin pH overlap with those from domestic mammals, except for some very alkaline (>9 pH) values in domestic sheep (likely because the wool was measured and not the skin; Supplementary Table S1). In humans, skin pH has a circadian rhythm in some, but not all, body parts and can vary ~0.3 pH, with maximal values in the afternoon (14:00–16:00) and minimal values in the evening (~20:00; Yosipovitch *et al.*, 1998). We measured captive bats during the day and wild bats during the night, except during hibernation when wild bats were also measured during the day, hence circadian rhythms may explain some of the variation we documented in bat skin pH.

We found that skin pH varied among bat species (Table 1; Fig. 3), which may be caused by multiple factors. Diet varies among the insectivorous bat species we studied. For example, *E. fuscus* may be beetle specialists (Thomas *et al.*, 2012) and captive *E. fuscus* in our study were exclusively fed meal worms (i.e. Tenebrionid larvae). In humans, there is contradictory evidence for the effect of diet on skin pH (Prakash *et al.*, 2017; Lim *et al.*, 2019), and skin pH in cattle and cats did not vary with diet (Jenkinson and Mabon, 1973; Bourdeau *et al.*, 2004). However, sebum can be affected by diet (Lovászi *et al.*, 2017). Sebum quantity and skin pH are inversely correlated in humans (Wan *et al.*, 2014), and bat flight membranes have sebaceous glands that vary in abundance by species (Cortese and Nicoll, 1970; Sokolov, 1982; Yin *et al.*, 2011). The composition and quantity of fatty acids that comprise sebum on bat flight membranes also varies among species and seasons (Pannkuk *et al.*, 2012; Frank *et al.*, 2016) and could affect skin pH. This may influence or be influenced by seasonal variation in skin pH given that enzyme activity in epidermal tissue, which produces fatty acids, is pH dependent (Behne *et al.*, 2002; Fluhr *et al.*, 2004a). Some free fatty acids are generated within skin from phospholipids by secretory phospholipase A_2_, and this enzyme is inactivated at alkaline pH (>7 pH), partially due to the activation of serine proteases (Behne *et al.*, 2002; Fluhr *et al.*, 2004b). The total fatty acid content of bat wing skin decreases over hibernation (Frank *et al.*, 2016), and we found that the skin of hibernating bats is typically alkaline. However, the skin pH of young laboratory mice with sebaceous gland hypoplasia did not differ from wild-type mice, suggesting minimal effect of sebaceous gland products on the development of adult acidic skin pH from the neonatal alkaline state (Fluhr *et al.*, 2004a). The acidification of neonate skin starts in deeper layers and moves upwards to the surface, and a pH gradient also exists in adults as deeper layers are more acidic compared to the skin surface (Behne *et al.*, 2002, 2003; Fluhr *et al.*, 2004a). This highlights the importance of endogenous skin processes in maintaining an acidic skin pH, such as the activity of the sodium-proton antiporter and secretory phospholipase A_2_ (Behne *et al.*, 2002, 2003; Fluhr *et al.*, 2004a). We acknowledge that humans and laboratory mice may not be the most relevant model systems for understanding skin chemistry in wild bats, but these studies provide evidence for drivers of skin pH, allowing us to generate testable hypotheses for future research in wild mammals. We also acknowledge that we only measured the surface pH of bat skin and that a pH gradient may exist within bat wing tissue like that observed in mice and humans. However, dermal and hypodermal layers of bat wings are greatly reduced compared to typical mammalian skin (Sokolov, 1982), suggesting lower variation than in other mammals. Finally, the current study does not allow us to untangle the associations among sebum, skin pH, and diet in bats, which should be further investigated.

In humans, cutaneous pH varies among body parts, and occluded areas (e.g. axillae, genitoanal region, submammary folds and interdigital areas) are generally more alkaline (6–7 pH) than drier areas (4–6 pH) (Schmid-Wendtner and Korting, 2006). Skin pH also varies among body parts in domestic mammals (Jenkinson and Mabon, 1973; Mok *et al.*, 1982; Meyer and Neurand, 1991; Ruedisueli *et al.*, 1998; Proksch, 2018). Roosting bats fold their wings, which may increase moisture and lead to higher pH levels. The uropatagium may be more acidic than the wing in bats (Fig. 3) because of repeated exposure to urine, an acidic liquid. The urine pH of the bat species we studied may be as acidic as *Myotis velifer* urine (mean: 5.5–6.0 pH, range: 5.1–9.4 pH; depending on month) (Shackelford and Caire, 1993) or that of 5 European bat species (mean: 5.3–6.8 pH) (Hales, 2014). In humans, grooming habits affect skin pH. For example, showering temporarily increases skin pH (Lambers *et al.*, 2006). Grooming (i.e. licking) skin may temporarily affect bat skin pH as *M. lucifugus* and *M. septentrionalis* saliva is ~7.5–8.5 pH (Dumont, 1997), and may partially explain why different body parts varied in pH if bats do not groom all areas equally. However, the pH of the roosting environment may also affect skin pH. Dorsal flight membrane surfaces may have been more acidic than ventral surfaces year-round in the captive *E. fuscus* colony because roosting substrates are acidic year-round (except for a short period after roost towels are cleaned/replaced). Roosting substrates in the captive *E. fuscus* colony were likely acidic due to the accumulation of body wastes, especially urine and new roosting towels became noticeably stiffer with time due to saturation with dried urine. This suggests interior surfaces of natural maternity roosts are also acidic due to accumulation of nitrogenous waste and dorsal flight membranes were more acidic than ventral surfaces during maternity season. During swarming and hibernation wild bats roost on cave walls, which are generally alkaline (7–8 pH) (Hajna, 2003; Shahack-Gross *et al.*, 2004; Portillo and Gonzalez, 2010), and their dorsal flight membranes were more alkaline than ventral surfaces during this time. Our findings indicate that roosting substrates within bat colonies may influence skin pH and therefore possibly skin function, which is relevant to experiments involving captive bat colonies.

The pH of roosting substrates affects skin pH and may partially explain the seasonal patterns we observed (Fig. 4), as bats switch roosts from one season to the next. However, we also documented seasonal variation in skin pH in the captive *E. fuscus* colony, where bats live in the same enclosure and therefore urinate on the same roosting substrates year-round. Seasonal variation in skin pH has also been documented in humans, many of which do not change dwellings seasonally (Abe *et al.*, 1980; Nakagawa *et al.*, 2004; Wan *et al.*, 2014). Humans are most acidic in July and most alkaline in January, although subjects were only measured four months of the year (Abe *et al.*, 1980). The mean seasonal change in human skin pH is 0.4–1.5 from summer to winter (Abe *et al.*, 1980; Nakagawa *et al.*, 2004; Wan *et al.*, 2014), while we documented a mean change of 2.1 and 1.4 pH from July to February in wild and captive bats, respectively. The larger seasonal variation in skin pH of wild bats is likely related to the effects of hibernation, exposure to outside temperatures, and changes in roosting substrates. Future studies should consider repeated measures from wild maternity colonies throughout the active season to help untangle the effects of site and season on skin pH.

Ambient temperature and humidity may partially drive seasonal changes in skin pH indirectly by affecting sweat and sebum production. We did not detect an effect of temperature or relative humidity on the skin pH of captive *E. fuscus*, likely because these bats were somewhat buffered from the seasonal changes in weather experienced by wild bats (Supplementary Fig. S1). One hypothesis for low skin pH during summer in humans is increased eccrine sweat secretion stimulated by increasing skin temperature (Abe *et al.*, 1980). This explanation is unlikely to apply to bats as eccrine glands in non-human mammals are confined to footpads (Folk and Semken, 1991), and instead bats evaporatively cool by panting or licking and fanning their wings (Baudinette *et al.*, 2000). Sweat glands in bats are reported as either absent (Sokolov, 1982; Makanya and Mortola, 2007) or exclusively apocrine (Sisk, 1957; Cortese and Nicoll, 1970). Sebum quantity and skin pH are inversely correlated in humans, and sebaceous gland activity increases with increases in humidity and especially temperature (Sakuma and Maibach, 2012; Wan *et al.*, 2014).

We found a sex difference in skin pH among bats, but only during the active season (Table 1; Fig. 3), possibly due to the use of different roost types by the sexes and/or hormonal variation. In many temperate insectivorous bats, the sexes largely segregate from early spring through mid-summer with females forming maternity colonies and males in bachelor groups (Kunz and Fenton, 2003). Skin pH is higher in males than females in dogs (Ruedisueli *et al.* 1998), cats (Szczepanik *et al.*, 2011) and cattle (Jenkinson and Mabon, 1973; Meyer and Neurand, 1991), although other studies on various domestic mammals found no difference between the sexes (Supplementary Table S1). In humans, there are conflicting results concerning which sex is more acidic (Giacomoni *et al.*, 2009).

Age affects skin pH as neonates (<1 month) and elderly humans (> 60 years) have more alkaline skin than adults, as do neonate laboratory rats and calves (Ajito *et al.*, 2001; Fluhr *et al.*, 2004a; Choi *et al.*, 2007; Chan and Mauro, 2011; Proksch, 2018). Rats attain adult skin pH levels ~1 week after birth (Fluhr *et al.*, 2004ab), and humans after ~1 month (Proksch, 2018). We found that volant juvenile bats had more acidic skin than adults (adults could not be aged) during maternity season but not during swarming (Table 1; Fig. 6). Potentially, this reflects the large amount of time juveniles spend in maternity roosts as these roosting substrates may be acidic due to waste accumulation (we only measured the pH of roosting substrates in the captive colony).

Variation in skin pH among species and individuals may impact susceptibility to skin diseases. For example, the relatively high skin pH of dogs (7–8 pH) compared to other domestic animals may partially account for the disproportionally high incidence of pyoderma (superficial bacterial infection of hair follicles and surrounding skin) in dogs (Mason *et al.*, 1996). Studies in humans, dogs, laboratory mice and rats indicate that experimentally decreasing skin pH with topical products can prevent or ameliorate some skin diseases and speed recovery from injury, but not in all circumstances (Matousek *et al.*, 2003; Fluhr *et al.*, 2004b) Hatano *et al.*, 2009; Lee *et al.*, 2014; Nagoba *et al.*, 2015; Panther and Jacob, 2015). In bats with WNS, it is unknown if the fungal pathogen (*P. destructans*) causes fewer skin lesions on acidic versus alkaline skin. Our data show that *E. fuscus* has the most acidic skin (Fig. 3), and this species is also more tolerant of WNS than the other bat species we measured (Cheng *et al.*, 2021). Although *P. destructans* can grow *in vitro* from 4.5–11 pH (Raudabaugh and Miller, 2013; Vanderwolf *et al.*, 2021), a carboxypeptidase enzyme produced by *P. destructans* was most active at 3–5 pH compared to 6.5–8.5 pH *in vitro* (Beekman *et al.*, 2018). The skin of wild bats during hibernation varied from 6.2 to 8.6 pH, suggesting that activity of this enzyme may be limited on the hibernating bats we measured. The activity of other potential virulence factors produced by *P. destructans*, the activity of bat skin defences such as cutaneous antimicrobial peptides, and potential biological or chemical spray-on treatments for WNS should be assessed at pH levels representative of the skin of hibernating bats of different species. For example, some yeasts commonly cultured from bat wings inhibit *P. destructans in vitro*, but only at 4–5 pH and not 7 pH (Vanderwolf *et al.*, 2021). This suggests that inhibition of *P. destructans* by these yeasts would not occur during hibernation on the skin of the bat species we measured during this study, since skin pH was >7 pH during winter (Fig. 3). However, pathogenic fungi can sense and respond to environmental pH, enabling survival, growth, virulence, and dissemination in different host niches by altering gene expression to produce enzymes that are functional at ambient pH (Martinez-Rossi *et al.*, 2017).

Future research on the influence of skin pH on the functionality of enzymes produced by microbes and bats may provide valuable insights on new therapeutic targets for treating bat skin conditions like WNS. Skin enzyme functionality is important in maintaining skin barrier function and for virulence factors produced by microbes. Skin pH may play a role in varying disease susceptibility among individuals and species by influencing enzyme functionality or the diversity of cutaneous microbiota. More data on skin pH in relation to other aspects of skin chemistry and from more bat species in different geographic areas may provide further insights on bat skin disease susceptibility.

## Supplementary material

Supplementary material is available at *Conservation Physiology* online.

## Funding

This work was supported by New Brunswick Department of Energy & Resource Development; New Brunswick Wildlife Trust Fund to K.J.V. and D.F.M. [B309-145]; Trent University; Ontario Ministry of Northern Development, Mines, Natural Resources and Forestry; Sigma Xi to K.J.V. [G201903158417906]; Natural Sciences and Engineering Research Council of Canada [Discovery Grants awarded to C. M. D. and P.A.F.]; and the New Brunswick Museum.

## Supplementary Material

Web_Material_coab088
