# Erratum to: Skin pH varies among bat species seasons and between wild and captive bats

**DOI:** 10.1093/conphys/coac003

**Published:** 2022-01-11

**Authors:** Karen J Vanderwolf, Christopher J Kyle, Paul A Faure, Donald F McAlpine, Christina M Davy

**Affiliations:** 1 Environmental and Life Sciences Program, Trent University, 1600 West Bank Dr., Peterborough, K9L 0G2, Ontario, Canada; 2 Department of Natural History, New Brunswick Museum, 277 Douglas Ave, Saint John, E2K 1E5, New Brunswick, Canada; 3 Forensic Science Department, Trent University, 1600 West Bank Dr, Peterborough, K9L 0G2, Ontario, Canada; 4 Natural Resources DNA Profiling and Forensics Center, Trent University, 1600 West Bank Dr, Peterborough, K9L 0G2, Ontario, Canada; 5 Department of Psychology, Neuroscience & Behaviour, McMaster University, 1280 Main Street West, Hamilton, L8S 4K1, Ontario, Canada; 6 Wildlife Research and Monitoring Section, Ontario Ministry of Northern Development, Mines, Natural Resources and Forestry, 1600 West Bank Dr, Peterborough, K9L 0G2, Ontario, Canada; 7 Department of Biology, Carleton University, 1125 Colonel By Drive, Ottawa, K1S 5B6, Ontario, Canada

*Conserv Physiol* 9(1): coab088; doi: 10.1093/conphys/coab088

In the originally published version of this manuscript, the incorrect Supplementary Material was included. This has been replaced with the correct information. The publisher apologizes for the error.

