# Supplementary material for: Skin pH varies among bat species and seasons and between wild and captive bats
Source: Conserv Physiol. 2021 Dec 6;9(1):coab088. doi: 10.1093/conphys/coab088 (PMC8672241; doi:10.1093/conphys/coab088)
Supplement: Web_Material_coab088 [file web_material_coab088.zip › Vanderwolf Supplemental.docx]

**Supplementary Materials**


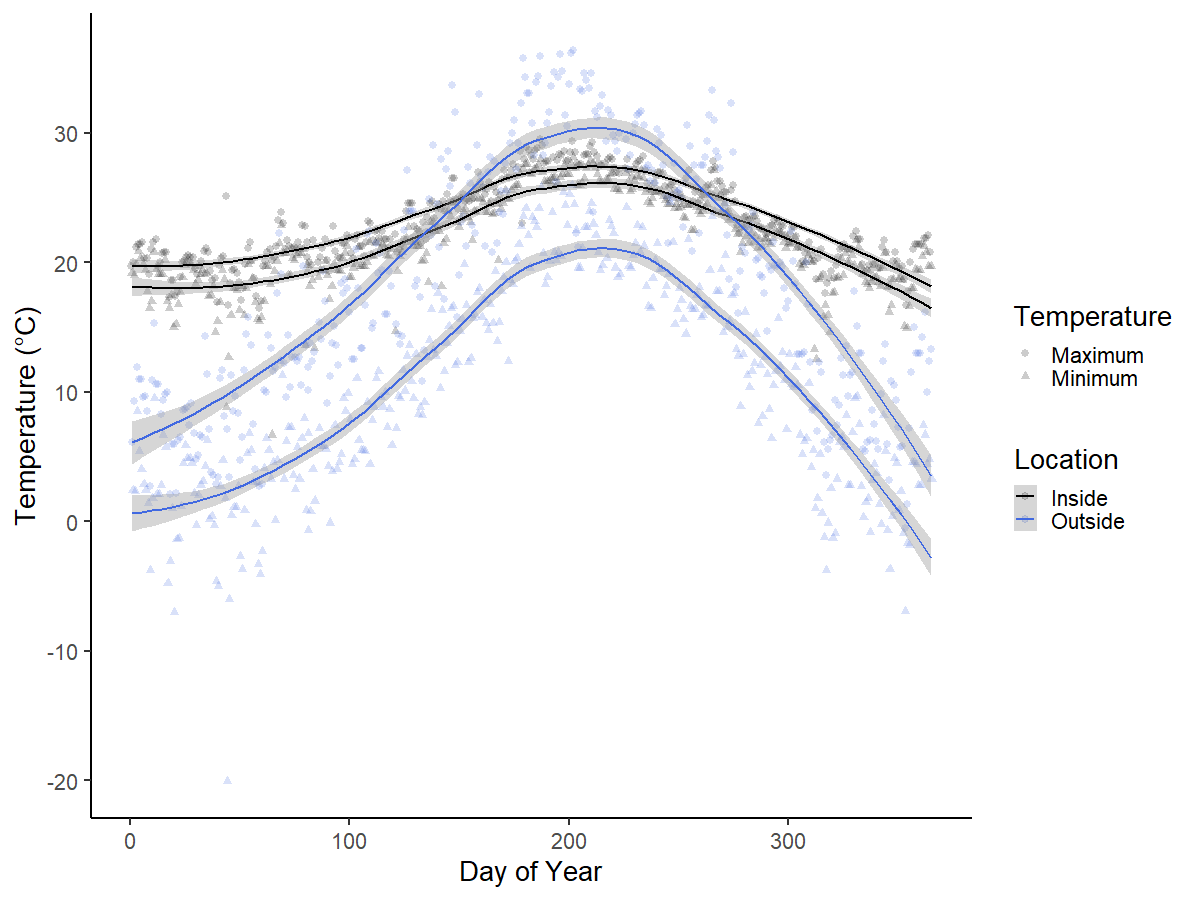


Figure S1: Daily maximum and minimum temperatures recorded April 2019 – March 2020 inside and outside the captive *Eptesicus fuscus* colony in Hamilton, Ontario. Markers represent individual data points while the lines indicate the mean with 95% confidence intervals in gray shading.


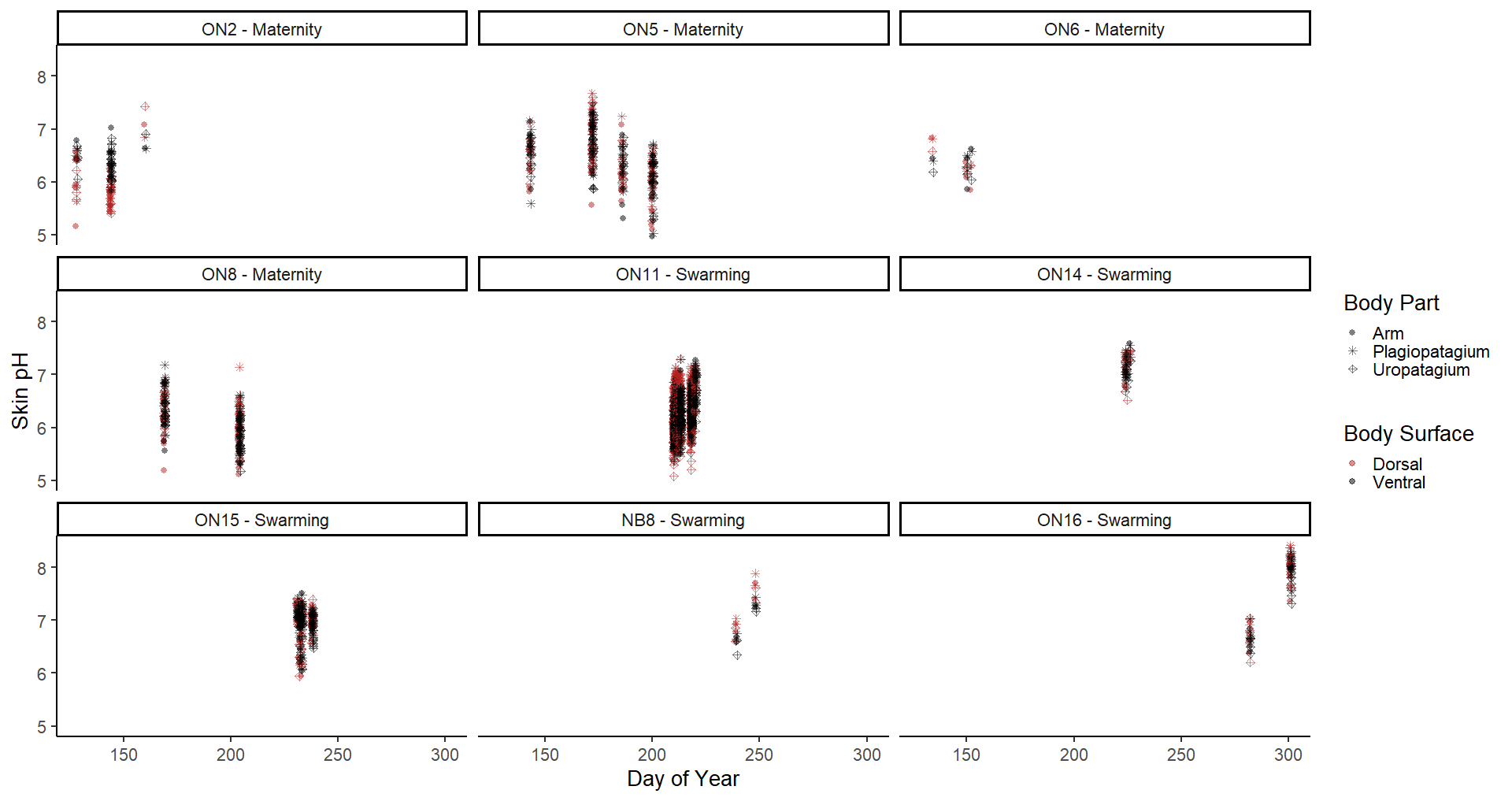


Figure S2: Sites where wild bats were sampled over multiple time points. Sites that were sampled only twice and with two days or less between visits were excluded.


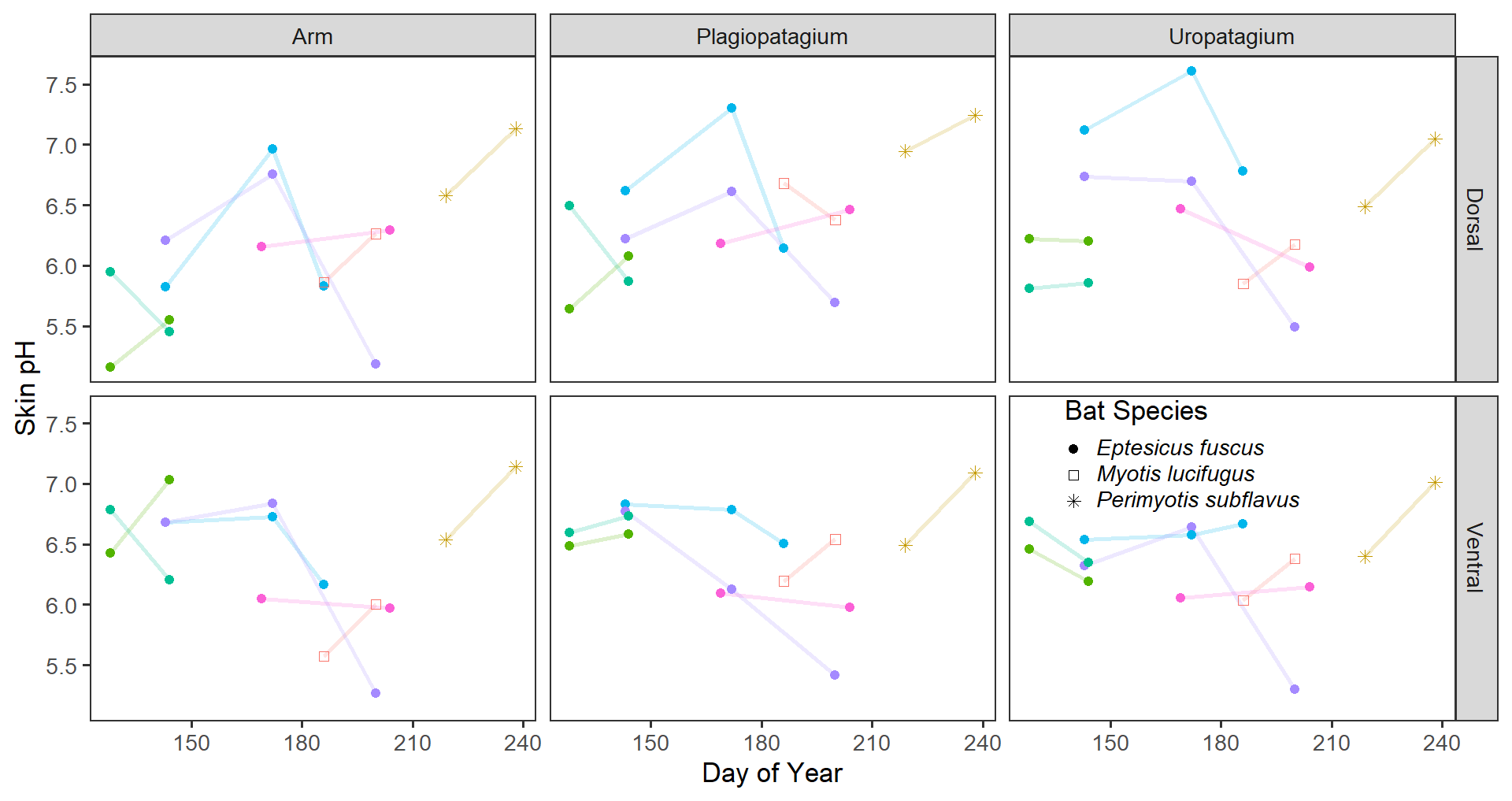


Figure S3: Skin pH of seven wild bats in Ontario that were captured two to three times over summer 2019. Each color indicates an individual bat.


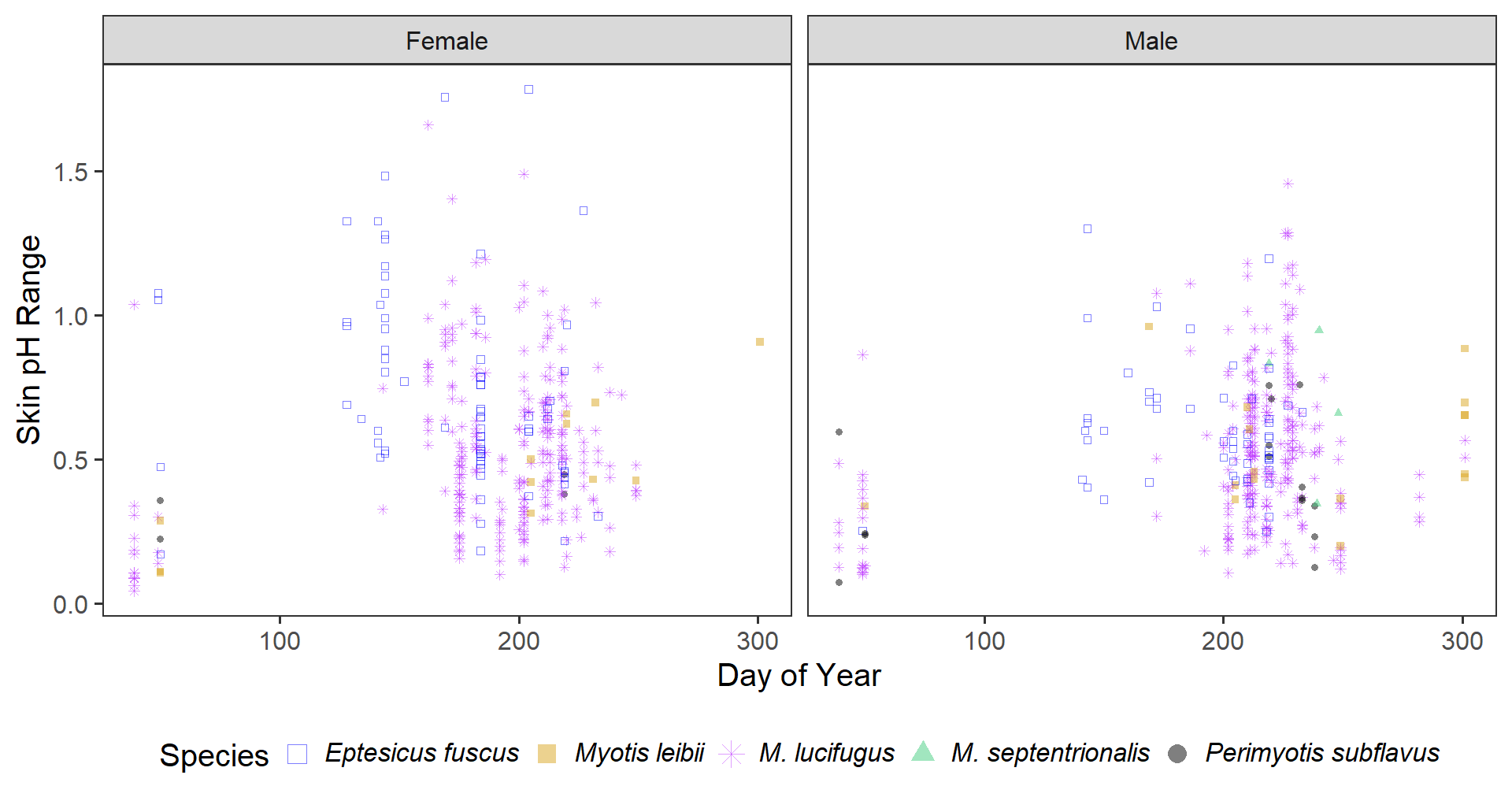


Figure S4: Range in skin pH values among the six body parts measured on individual wild bats. Range was calculated by subtracting the lowest from the highest value on each bat.

Table S2: Sample sizes for each bat species in each month. The province where bats were measured is indicated after the sample size O=Ontario, N= New Brunswick, P=Prince Edward Island, Q=Quebec. Captive *Eptesicus fuscus* were sampled in Hamilton, Ontario. F = female and M = male.

| **Species** | **Captive *E. fuscus*** | | **Wild *E. fuscus*** | | ***Myotis lucifugus*** | | ***M. leibii*** | | ***M. septentrionalis*** | | ***Perimyotis subflavus*** | |
| --- | --- | --- | --- | --- | --- | --- | --- | --- | --- | --- | --- | --- |
| **Sex** | **F** | **M** | **F** | **M** | **F** | **M** | **F** | **M** | **F** | **M** | **F** | **M** |
| January | 53 | 24 |  |  |  |  |  |  |  |  |  |  |
| February | 58 | 24 | 4 Q | 1 Q | 13 O, 3 Q | 5 O, 15 Q | 3 Q | 1 Q |  |  | 2 Q | 2 O, 2 Q |
| March | 63 | 23 |  |  |  |  |  |  |  |  |  |  |
| April | 8 | 18 |  |  |  |  |  |  |  |  |  |  |
| May | 16 | 10 | 23 O | 10 O | 2 O |  |  |  |  |  |  |  |
| June | 15 | 6 | 3 O | 7 O | 29 O, 35 P | 3 O |  | 1 O |  |  |  |  |
| July | 16 | 9 | 9 O, 27 N | 19 O | 76 O, 32 N | 86 O, 2 N | 3 O | 4 O |  |  |  |  |
| August | 39 | 14 | 10 O, 1 N | 16 O, 1 N | 59 O, 6 N | 74 O, 66 N | 4 O | 2 O |  | 1 O, 2 N | 2 O | 11 O |
| September | 63 | 25 |  |  | 4 O | 10 O, 2 N | 1 O | 2 O |  | 1 N |  |  |
| October | 60 | 26 |  |  |  | 6 O | 1 O | 6 O |  |  |  |  |
| November | 40 | 20 |  |  |  |  |  |  |  |  |  |  |
| December | 53 | 20 |  |  |  |  |  |  |  |  |  |  |
| **Total** | **484** | **219** | **77** | **54** | **259** | **269** | **12** | **16** | **0** | **4** | **4** | **15** |

Table S1: Summary of previous research on the skin pH of non-human vertebrates. 'Months' indicates when measurements were taken. Whether the hair/fur of animals were shaved or clipped prior to measurement is indicated, although some animals and neonates are naturally hairless. When available, pH ranges are given in brackets in the 'mean pH' column. Articles on the skin pH of laboratory mice are representative of a larger literature body (Draize, 1942; Roy, 1954; Grono, 1970; Jenkinson and Mabon, 1973; Bartels *et al.*, 1991; Meyer and Neurand, 1991; Meyer *et al.*, 1991, 2001; Bogacz, 1992; Mauro *et al.*, 1998; Ruedisueli *et al.*, 1998; Ajito *et al.*, 2001; Fluhr *et al.*, 2001; Dunstan *et al.*, 2002; Hamann *et al.*, 2002; Behne *et al.*, 2002, 2003; Tsui *et al.*, 2002; Young *et al.*, 2002; Fox *et al.*, 2003; Hachem *et al.*, 2003, 2005, 2010; Matousek *et al.*, 2003; J. W. Fluhr *et al.*, 2004; J.W. Fluhr *et al.*, 2004; Popiel and Nicpoń, 2004; Bourdeau *et al.*, 2004; Zecconi *et al.*, 2005; Litwiller *et al.*, 2006; Choi *et al.*, 2007; Hatano *et al.*, 2009; Oh and Oh, 2009; Ferreira, 2010; Pan *et al.*, 2010; Szczepanik *et al.*, 2011, 2012, 2013; Breathnach *et al.*, 2011; Woodhams *et al.*, 2012; Moniaga *et al.*, 2013; Danciu *et al.*, 2014; Gołyński *et al.*, 2014; Lee *et al.*, 2014, 2016; Sakai *et al.*, 2014; Zajac *et al.*, 2015; Jang *et al.*, 2016; Bradley *et al.*, 2016; Hobi *et al.*, 2017; Koziol *et al.*, 2017; Mašínová *et al.*, 2017; Joly, 2018; Klinger *et al.*, 2018; Urnau, 2018; Cobiella *et al.*, 2019; Menon *et al.*, 2019; Proksch and Neumann, 2019; Barnhart *et al.*, 2020; Santoro *et al.*, 2021b, 2021a; Tang *et al.*, 2021; Wen *et al.*, 2021).

**References**

Ajito T, Suzuki K, Okumura J, Hatano N (2001) Skin pH of domestic animals. *Japanese J Vet Clin* 24: 9–12.

Barnhart K, Bletz MC, LaBumbard B, Tokash-Peters A, Gabor CR, Woodhams DC (2020) *Batrachochytrium salamandrivorans* elicits acute stress response in spotted salamanders but not infection or mortality. *Anim Conserv* 23: 533–546.

Bartels T, Meyer W, Neurand K (1991) Comparative study on the surface pH of avian skin. *J Ornithol* 132: 279–284.

Behne MJ, Barry NP, Hanson KM, Aronchik I, Clegg RW, Gratton E, Feingold K, Holleran WM, Elias PM, Mauro TM (2003) Neonatal development of the stratum corneum pH gradient: localization and mechanisms leading to emergence of optimal barrier function. *J Invest Dermatol* 120: 998–1006.

Behne MJ, Meyer JW, Hanson KM, Barry NP, Murata S, Crumrine D, Clegg RW, Gratton E, Holleran WM, Elias PM, *et al.* (2002) NHE1 regulates the stratum corneum permeability barrier homeostasis: Microenvironment acidification assessed with fluorescence lifetime imaging. *J Biol Chem* 277: 47399–47406.

Bogacz E (1992) Próbo określenio pH błon śluzowych i powierzchni skóry owiec. *Med Weter* 48: 114–115.

Bourdeau P, Taylor KW, Nguyen P, Biourge V (2004) Evaluation of the influence of sex, diet and time on skin pH and surface lipids of cats. *Vet Dermatol* 15: 41–69.

Bradley CW, Morris DO, Rankin SC, Cain CL, Misic AM, Houser T, Mauldin EA, Grice EA (2016) Longitudinal evaluation of the skin microbiome and association with microenvironment and treatment in canine atopic dermatitis. *J Invest Dermatol* 136: 1182–1190.

Breathnach RM, Quinn PJ, Baker KP, Mcgeady T, Abbott Y, Jones BR (2011) Association between skin surface pH, temperature, and *Staphylococcus pseudintermedius* in dogs with immunomodulatory-responsive lymphocytic-plasmacytic pododermatitis. *Vet Dermatol* 22: 312–318.

Choi E, Man M, Xu P, Xin S, Liu Z, Crumrine DA, Jiang YJ, Fluhr JW, Feingold KR, Elias PM, *et al.* (2007) Stratum corneum acidification is impaired in moderately aged human and murine skin. *J Insect Conserv* 127: 2847–2856.

Cobiella D, Archer L, Bohannon M, Santoro D (2019) Pilot study using five methods to evaluate skin barrier function in healthy dogs and in dogs with atopic dermatitis. *Vet Dermatol* 30: 121–126.

Danciu C, Coricovac DE, Soica C, Dumitrascu V, Simu G, Antal D, Lajos K, Dehelean CA, Borcan F (2014) Evaluation of skin physiological parameters in SKH1 mice experimental model after exposure to aggressive factors like UVB using non-invasive methods. *Rev Chim* 65: 1195–1199.

Draize JH (1942) The determination of the pH of the skin of man and common laboratory animals. *J Invest Dermatol* 5: 77–85.

Dunstan R, Herdt T, Olivier B, Mei B, Credille K, Kennis R, Maier R, Olivier B, Castle S, Reinhart G, *et al.* (2002) Age- and breed-related differences in canine skin surface lipids and pH. In: Thoday K, Foil C, Bond R, eds. Advances in Veterinary Dermatology, Volume 4. Proceedings of the Fourth World Congress of Veterinary Dermatology. Blackwell Science, San Francisco, California, pp 37–42.

Ferreira D (2010) Estudo Do PH Da Pele Em Cães Saudáveis e Cães Com Insuficiência Renal Crónica. Technical University of Lisbon.

Fluhr J. W., Behne MJ, Brown BE, Moskowitz DG, Selden C, Mao-Qiang M, Mauro TM, Elias PM, Feingold KR (2004) Stratum corneum acidification in neonatal skin: secretory phospholipase A2 and the sodium/hydrogen antiporter-1 acidify neonatal rat stratum corneum. *J Invest Dermatol* 122: 320–329.

Fluhr JW, Kao J, Jain M, Ahn SK, Feingold KR, Elias PM (2001) Generation of free fatty acids from phospholipids regulates stratum corneum acidification and integrity. *J Invest Dermatol* 117: 44–51.

Fluhr J.W., Mao-Qiang M, Brown BE, Hachem JP, Moskowitz DG, Demerjian M, Haftek M, Serre G, Crumrine D, Mauro TM, *et al.* (2004) Functional consequences of a neutral pH in neonatal rat stratum corneum. *J Invest Dermatol* 123: 140–151.

Fox LK, Oura LY, Ames CR (2003) Short communication: teat skin pH. *J Dairy Sci* 86: 3951–3952.

Gołyński M, Szczepanik M, Lutnicki K, Adamek Ł, Gołyńska M, Wilkołek P, Sitkowski W, Kurek Ł, Dębiak P (2014) Biophysical parameters of rats ’ skin after the administration of methimazole. *Bull Vet Inst Pulawy* 58: 315–319.

Grono L (1970) Studies of the microclimate of the external auditory canal in the dog: II. Hydrogen ion concentration of the epithelial surface of the external auditory meatus. *Res Vet Sci* 11: 312–315.

Hachem J, Crumrine D, Fluhr J, Brown B, Feingold K, Elias P (2003) pH directly regulates epidermal permeability barrier homeostasis, and stratum corneum integrity/cohesion. *J Invest Dermatol* 121: 345–353.

Hachem J, Man M, Crumrine D, Uchida Y, Brown B, Rogiers V, Roseeuw D, Feingold K, Elias P (2005) Sustained serine proteases activity by prolonged increase in pH leads to degradation of lipid processing enzymes and profound alterations of barrier function and stratum corneum integrity. *J Invest Dermatol* 125: 510–520.

Hachem J, Roelandt T, Schürer N, Pu X, Fluhr J, Man M, Crumrine D, Roseeuw D, Feingold KR, Mauro T, *et al.* (2010) Acute acidification of stratum corneum membrane domains using polyhydroxyl acids improves lipid processing and inhibits degradation of corneodesmosomes. *J Invest Dermatol* 130: 500–510.

Hamann J, Hansen S, Lacy-Hulbert SJ, Woolford M (2002) Measurement of bovine teat skin pH. *Milchwissenschaft* 57: 490–493.

Hatano Y, Man M, Uchida Y, Crumrine D, Scharschmidt TC, Kim EG, Mauro TM, Feingold KR, Elias PM, Holleran WM (2009) Maintenance of an acidic stratum corneum prevents emergence of murine atopic dermatitis. *J Invest Dermatol* 129: 1824–1835.

Hobi S, Klinger C, Classen J, Mueller RS (2017) The effects of a topical lipid complex therapy on dogs with atopic dermatitis: a double blind, randomized, placebo-controlled study. *Vet Dermatol* 28: 369-e84.

Jang H, Matsuda A, Jung K, Karasawa K, Matsuda K, Oida K, Ishizaka S, Ahn G, Amagai Y, Moon C, *et al.* (2016) Skin pH is the master switch of kallikrein 5-mediated skin barrier destruction in a murine atopic dermatitis model. *J Invest Dermatol* 136: 127–135.

Jenkinson MD, Mabon RM (1973) The effect of temperature and humidity on skin surface pH and the ionic composition of skin secretions in Ayrshire cattle. *Br Vet J* 129: 282–295.

Joly G (2018) Avaliacao de PH Cutaneo de Caes Da Raca Shih Tzu Com Dermatite Atopica. Universidade do sul de Santa Catarina.

Klinger CJ, Hobi S, Johansen C, Koch H, Weber K, Mueller RS (2018) Vitamin D shows in vivo efficacy in a placebo-controlled, double-blinded, randomised clinical trial on canine atopic dermatitis. *Vet Rec* 182: 406.

Koziol J, Fraser N, Passler T, Wolfe D (2017) Initial steps in defining the environment of the prepuce of the bull by measuring pH and temperature. *Aust Vet J* 95: 480–482.

Lee HJ, Yoon NY, Lee NR, Jung M, Kim DH, Choi EH (2014) Topical acidic cream prevents the development of atopic dermatitis- and asthma-like lesions in murine model. *Exp Dermatol* 23: 736–741.

Lee NR, Lee H, Yoon NY, Kim D, Jung M, Choi EH (2016) Application of topical acids improves atopic dermatitis in murine model by enhancement of skin barrier functions regardless of the origin of acids. *Ann Dermatol* 28: 690–696.

Litwiller SL, O’Donnell MJ, Wright PA (2006) Rapid increase in the partial pressure of NH3 on the cutaneous surface of air-exposed mangrove killifish, *Rivulus marmoratus*. *J Exp Biol* 209: 1737–1745.

Mašínová T, Pontes A, Carvalho C, Sampaio JP, Baldrian P (2017) *Libkindia masarykiana* gen. et sp. nov., *Yurkovia mendeliana* gen. et sp. nov. and *Leucosporidium krtinense* f.a. sp. nov., isolated from temperate forest soils. *Int J Syst Evol Microbiol* 67: 902–908.

Matousek J, Campbell K, Kakoma I, Schaeffer D (2003) The effects of four acidifying sprays, vinegar, and water on canine cutaneous pH levels. *J Am Anim Hosp Assoc* 39: 29–33.

Mauro T, Grayson S, Gao WN, Man MQ, Kriehuber E, Behne M, Feingold KR, Elias PM (1998) Barrier recovery is impeded at neutral pH, independent of ionic effects: implications for extracellular lipid processing. *Arch Dermatol Res* 290: 215–222.

Menon GK, Catania KC, Crumrine D, Bradley C, Mauldin EA (2019) Unique features of the skin barrier in naked mole rats reflect adaptations to their fossorial habitat. *J Morphol* 280: 1871–1880.

Meyer W, Neurand K (1991) Comparison of skin pH in domesticated and laboratory mammals. *Arch Dermatol Res* 283: 16–18.

Meyer W, Neurand K, Bartels T (1991) The acid mantle of the skin of domesticated animals. *Dtsch Tierarztl Wochenschr* 98: 167–170.

Meyer W, Neurand K, Tanyolac A (2001) General anti-microbial properties of the integument in fleece producing sheep and goats. *Small Rumin Res* 41: 181–190.

Moniaga CS, Jeong SK, Egawa G, Nakajima S, Hara-Chikuma M, Jeon JE, Lee SH, Hibino T, Miyachi Y, Kabashima K (2013) Protease activity enhances production of thymic stromal lymphopoietin and basophil accumulation in flaky tail mice. *Am J Pathol* 182: 841–851.

Oh W, Oh T (2009) Mapping of the dog skin based on biophysical measurements. *Vet Dermatol* 21: 367–372.

Pan T, Wang P, Lee W, Fang C, Chen C, Huang C, Fang J (2010) Systematic evaluations of skin damage irradiated by an erbium: YAG laser: histopathologic analysis, proteomic profiles, and cellular response. *J Dermatol Sci* 58: 8–18.

Popiel J, Nicpoń J (2004) Relacje pomiędzy ph skóry w przebiegu pyoderm u psów przed i po zastosowaniu preparatów działających zewnętrznie. *Acta Sci Pol Med Vet* 3: 53–60.

Proksch E, Neumann C (2019) Influence of buffers of different pH and composition on the murine skin barrier, epidermal proliferation, differentiation, and inflammation. *Skin Pharmacol Physiol* 32: 328–336.

Roy WE (1954) Role of the sweat glands in eczema of dogs: a preliminary report. *J Am Vet Med Assoc* 124: 51–54.

Ruedisueli F, Eastwood N, NK G, Watson T (1998) The measurement of skin pH in normal dogs of different breeds. In: Kwochka K, Willemse T, Von Tscharner C, eds. Advances in Veterinary Dermatology, Vol 3. Edition. Butterworth-Heineman, Oxford, pp 521–522.

Sakai T, Hatano Y, Zhang W, Fujiwara S (2014) Defective maintenance of pH of stratum corneum is correlated with preferential emergence and exacerbation of atopic-dermatitis-like dermatitis in flaky-tail mice. *J Dermatol Sci* 74: 222–228.

Santoro D, Archer L, Fagman L (2021a) Intradermal immunotherapy with actinomycetales preparations as treatment for feline atopic syndrome: a randomized, placebo-controlled, double-blinded study. *Vet Dermatol* 8.

Santoro D, Fagman L, Zhang Y, Fahong Y (2021b) Clinical efficacy of spray-based heat-treated lactobacilli in canine atopic dermatitis: a preliminary, open-label, uncontrolled study. *Vet Dermatol* 32: 114–118.

Szczepanik MP, Wilkołek PM, Adamek ŁR, Pomorski ZJH (2011) The examination of biophysical parameters of skin (transepidermal water loss, skin hydration and pH value) in different body regions of normal cats of both sexes. *J Feline Med Surg* 13: 224–230.

Szczepanik MP, Wilkołek PM, Pluta M, Adamek ŁR, Gołyński M, Pomorski ZJH, Sitkowski W (2013) The examination of biophysical skin parameters (transepidermal water loss, skin hydration and pH value) in different body regions in Polish ponies. *Pol J Vet Sci* 16: 741–747.

Szczepanik MP, Wilkołek PM, Pluta M, Adamek R, Pomorski ZJH (2012) The examination of biophysical parameters of skin (transepidermal water loss, skin hydration and pH value) in different body regions of ponies. *Pol J Vet Sci* 15: 553–559.

Tang L, Cao X, Li X, Ding H (2021) Topical application with conjugated linoleic acid ameliorates 2, 4-dinitrofluorobenzene-induced atopic dermatitis-like lesions in BALB/c mice. *Exp Dermatol* 30: 237–248.

Tsui TKN, Randall DJ, Chew SF, Jin Y, Wilson JM, Ip YK (2002) Accumulation of ammonia in the body and NH3 volatilization from alkaline regions of the body surface during ammonia loading and exposure to air in the weather loach *Misgurnus anguillicaudatus*. *J Exp Biol* 205: 651–659.

Urnau L (2018) Avaliacao Do PH Da Pele de Caes Saudaveis Das Racas Golden Retriever e Shih Tzu. Universidade do sul de Santa Catarina.

Wen S, Wu J, Ye L, Yang B, Hu L, Man MQ (2021) Topical applications of a heparinoid-containing product attenuate glucocorticoid-induced alterations in epidermal permeability barrier in mice. *Skin Pharmacol Physiol* 34: 86–93.

Woodhams DC, Geiger CC, Reinert LK, Rollins-Smith LA, Lam B, Harris RN, Briggs CJ, Vredenburg VT, Voyles J (2012) Treatment of amphibians infected with chytrid fungus: Learning from failed trials with itraconazole, antimicrobial peptides, bacteria, and heat therapy. *Dis Aquat Organ* 98: 11–25.

Young LA, Dodge JC, Guest KJ, Cline JL, Kerr WW (2002) Age, breed, sex and period effects on skin biophysical parameters for dogs fed canned dog food. *J Nutr* 132: 1695S-1697S.

Zajac M, Szczepanik MP, Wilkolek P, Adamek L, Pomorski ZJH, Sitkowski W, Golynski M (2015) Assessment of a correlation between canine atopic dermatitis extent and severity index (CADESI-03) and selected biophysical skin measures (skin hydration, pH, and erythema intensity) in dogs with naturally occurring atopic dermatitis. *Can J Vet Res* 79: 136–140.

Zecconi A, Binda E, Dapra V, Hemling T, Piccinini R (2005) Field study on protocols for evaluation of teat skin conditions. *J Vet Med Ser B* 52: 219–225.
